# An audit of root canal filling quality performed by undergraduate pre-clinical dental students, Yemen

**DOI:** 10.1186/s12909-019-1798-1

**Published:** 2019-09-13

**Authors:** Mokhtar Saleh Al-Anesi, Mohammed M. AlKhawlani, Abdulaziz A. Alkheraif, Abdulghani Ali Al-Basmi, Mohammed Nasser Alhajj

**Affiliations:** 1 Conservative Department, Faculty of Dentistry, Thamar University, Dhamar, Yemen; 2 Dental Biomaterials Research Chair, Dental Health Department, College of Applied Medical Sciences, King Saud University, Riyadh, Kingdom of Saudi Arabia; 3 Private Dental Clinic, Dhamar, Yemen; 4 Department of Prosthodontics, Faculty of Dentistry, Thamar University, Dhamar, Yemen

## Abstract

**Background:**

Dental students are future dentists. Continuous assessment and improving of the educational curricula will ensure excellent academic performance of dental students and thus providing the community with the best treatment modalities. The aim of this study was to evaluate the root canal filling quality performed in extracted teeth by preclinical undergraduate Yemeni dental students.

**Methods:**

Root canal treatment was performed by undergraduate preclinical dental students on 331 extracted human teeth including 741 roots. The teeth were then collected and evaluated radiographically based on three criteria of quality (length, density, and taper). Cohen’s Kappa test was used to assess the agreement between the examiners and Chi-squared test was used for the association between the study variables. The level of significant was set at α < 0.05.

**Results:**

The results of the study revealed that the overall quality of roots canals fillings was poor. However, more than half of the study sample (53.4%) had adequate length, 13.1% had adequate density, and 14.2% had adequate taper. Anterior as well as single-rooted teeth had significantly better quality than posterior and multi-rooted teeth, respectively. The root canal fillings quality mandibular teeth was better than of maxillary teeth with no significant difference (*P* > 0.05).

**Conclusion:**

The findings of the study emphasize the need of improving the endodontic course in the preclinical level and more advanced techniques and instruments should be incorporated.

## Background

The aim of root canal treatment (RCT) is to mechanically and chemically clean, shape, and three dimensionally fill the root canal system to prevent periapical inflammation and to provide a good environment for healing of any apical inflammation. Practice of preclinical students on extracted teeth has been a universal method of teaching endodontics and giving students the opportunity of gaining expertise before working inside the patient’s mouth. According to the European Society of Endodontology, performing root canal treatment is a mandatory educational requirement for the training of dental students [1–3]. Different instruments (e.g.: stainless steel hand files, NiTi hand files, engine-driven NiTi, and rotary instruments), solutions for irrigation (e.g.: sodium hypochlorite, chlorhexidine, and hydrogen peroxide), sealers (e.g.: zinc oxide-eugenol, epoxy resin based, calcium hydroxide, and glass ionomer), obturation techniques (e.g.: lateral condensation, vertical compaction, carrier-based techniques, continuous wave technique, thermo-plasticized injection, and thermo-mechanical compaction) have been proposed for root canal treatment [4–6]. The evaluation of technical quality of the root canal treatment at this stage is based mainly on radiographical methods [7, 8].

In 2006, The European Society of Endodontology issued a consensus report stating guidelines for radiographic evaluation of root canal fillings “the prepared root canal space should be filled completely with no space between filling and canal wall. The root canal filling should be placed within 0.5–2 mm of the radiographical apex to prevent post-treatment disease” [9]. Similarly, the American Association of Endodontists in 2009 stated that “for radiographic evaluation of root canal fillings, the three qualities that should be observed are: length, shape and density. The length of an ideal fill should be from the canal’s apical minor constriction to the canal orifice unless a post is planned, the shape of the completed case is somewhat dependent on the instrumentation technique being used, and voids should not be visible on the radiographic image” [10].

Despite the importance of performing preclinical RCT, few studies regarding the evaluation of technical quality of preclinical students have been conducted [11–15]. The regular assessment of dental students’ outcomes in endodontics at the preclinical level will help to improve dental education as well as improving the quality of students’ clinical performance [16]. In Yemen, Madfa et al. [17] evaluated the quality of RCT performed by general dental practitioners and found that the RCT quality was poor. On the other hand, no previous studies in Yemen has been conducted to evaluate the quality of RCT performed by dental students. Dental students at the Faculty of Dentistry-Thamar University take the preclinical annual endodontic course at the fourth year of their 5-year bachelor program. During this year, students are trained to perform RCT on extracted human teeth. The aim of this study was to evaluate the technical quality of preclinical undergraduate students in endodontics at the Faculty of Dentistry, Thamar University, Yemen.

## Methods

This study was approved by the Research Ethics Committee of the Faculty of Dentistry, Thamar University; and the dental students gave their consent to publish their results. The Research Ethics Committee members exempted the study from the ethical approval as the teeth were basically indicated from extraction (orthodontic or periodontal reasons) and were used mainly for educational purpose. A total of 331 extracted teeth were evaluated after performing RCT by preclinical students at Thamar University, Yemen during the academic year 2016–2017. Teeth with complex configuration (fused root, merged canals, and C-shaped canals), external or internal resorption, open apices, calcified canals, and teeth sever curved roots were excluded. All extracted teeth were embedded by roots and fixed in acrylic teaching model. Access to pulp chamber was achieved using carbide round burs. The remaining pulpal tissues were removed and the working length was determined. Step-back technique was followed for root canal preparation using hand instrument stainless steel files of 0.02 taper (K-files, DiaDent Corp., Chungcheongbuk-do, Korea), and a solution of 1% sodium hypochlorite (NaOCl) was used for irrigation. All root canals were filled using cold lateral condensation technique with gutta-percha points of 0.02 taper (Sure-Endo, SureDent Corp., Gyeonggi-do, Korea) and zinc oxide eugenol (ZOE) sealer (Zinconol, Prevest DenPro, Jammu, India). Nevertheless, hand instrumentation with step-back technique may affect the quality of the RCT as it might result in some procedural errors such as: ledges, perforations, or canal transportation. Some of these intervening factors could be controlled by using NiTi rotary system. The postoperative radiographs were evaluated to assess the technical quality of the RCTs. Two experienced endodontists examined the radiographs independently using an X-ray viewer. The evaluation criteria were length, taper and density of root canal obturation. These criteria were based on the recommendations of the American Endodontics Association for the evaluation of RCT quality and used by some other studies [9, 11, 18–20]. Each parameter was scored as 0 or 1 as shown in Table 1. Considering that the failure in one criterion means failure of the treatment as well as the failure of one root in multi-rooted teeth will result in failure of the whole treatment, the overall score of RCT quality was calculated based on accepted evaluation (score 1) of the three criteria for one-rooted teeth or for all roots in multi-rooted teeth. For example, the overall quality of maxillary first molar with three roots was considered accepted when each root got score 1 in each evaluation criterion which means that each root should get a total score of 3 and the tooth should get a total score of 9. Cohen’s Kappa test was used to assess the agreement between the examiners. The results revealed almost perfect agreement (Kappa value > 0.85; *P* < 0.001) for all evaluation criteria [21]. The data were then statistically analyzed using SPSS software program for Windows version 25.0 (SPSS, Inc., Chicago, IL, USA). Chi-square analysis of association was used between the study variables. Level of significant was set at *P*-value < 0.05.

**Table 1:** Criteria used for radiographic quality assessment of root canal fillings

Parameters	Criteria	Definition	Score
Length of root canal filling	Adequate	Root filling ending 0–2 mm of the radiographic apex	1
	Over-filling	Root filling ending beyond the radiographic apex	0
	Short-filling	Root filling ending > 2 mm short of the radiographic apex	0
Density of root canal filling	Adequate	No voids present in the root filling or between root filling and root canal walls	1
	Inadequate	Voids present in the root filling or between root filling and root canal walls	0
Taper of root canal filling	Adequate	Consistent taper from the orifice to the apex	1
	Inadequate	No consistent taper from the orifice to the apex	0

## Results

A total of 331 teeth (180 (54.4%) maxillary teeth and 151 (45.6%) mandibular teeth) including 741 canals were investigated in this study. The most investigated teeth were mandibular first molars (27.8%) while, the most investigated canals were mesiobuccal canals (25.2%), and teeth with one canal represented only 14.6% of the total sample. Samples distribution are shown in Table 2.

**Table 2:** Distribution of the study sample according to location of jaw, location of tooth, number of roots, type of tooth, and location of canal

	N	%
Location of jaw		
Maxilla	180	54.4
Mandible	151	45.6
Location of tooth		
Anterior	108	32.6
Posterior	223	67.2
Number of roots		
Single-rooted teeth	108	32.6
Multi-rooted teeth	223	67.4
Type of tooth		
Upper central	32	9.7
Upper lateral	6	1.8
Upper canine	19	5.7
Upper 1st premolar	31	9.4
Upper 1st molar	86	26.0
Upper 2nd molar	6	1.8
Lower central	51	15.4
Lower 1st molar	92	27.8
Lower 2nd molar	8	2.4
Location of canal		
Mesial	5	0.7
Distal	100	13.5
Palatal	123	16.6
Buccal	31	4.2
One canal	108	14.6
Mesiolingual	95	12.8
Mesiobuccal	187	25.2
Distobuccal	92	12.4

The quality of the obturated canals revealed that more than half of the study sample (53.4%) were adequate in length while, only 13.1% were adequate in density and 14.2% were adequate in taper (Table 3). Comparison between maxilla and mandible showed non-significant differences in the three criteria of quality (*P* > 0.05). However, comparison according to location (anterior and posterior) or number of roots (single-rooted teeth and multi-rooted teeth) revealed that anterior as well as single-rooted teeth had better significant quality than posterior and multi-rooted teeth, respectively (Table 4).

**Table 3:** Quality of root canal fillings based on length, density, and taper criteria

		Frequency	Percent
Length	Inadequate	345	46.6
	Adequate	396	53.4
Density	Inadequate	644	86.9
	Adequate	97	13.1
Taper	Inadequate	636	85.8
	Adequate	105	14.2

**Table 4:** Quality of root canal fillings according to jaw, location, and number of roots

	Length		Density		Taper	
	Inadequate	Adequate	Inadequate	Adequate	Inadequate	Adequate
Maxilla	97 (53.9)	83 (46.1)	162 (90.0)	18 (10.0)	161 (89.4)	19 (10.6)
Mandible	73 (48.3)	78 (51.7)	129 (85.4)	22 (14.6)	124 (82.1)	27 (17.9)
	***P*** **= 0.323**		***P*** **= 0.237**		***P*** **= 0.058**	
Anterior	20 (18.5)	88 (81.5)	83 (76.9)	25 (23.1)	71 (65.7)	37 (34.3)
Posterior	150 (67.3)	73 (32.7)	208 (93.3)	15 (6.7)	214 (96.0)	9 (4.0)
	**P < 0.001**		**P < 0.001**		**P < 0.001**	
Single-rooted teeth	20 (18.5)	88 (81.5)	83 (76.9)	25 (23.1)	71 (65.7)	37 (34.3)
Multi-rooted teeth	150 (67.3)	73 (32.7)	208 (93.3)	15 (6.7)	214 (96.0)	9 (4.0)
	**P < 0.001**		**P < 0.001**		***P*** **< 0.002**	

Boldface refers to significance at *P* value < 0.05

In the maxilla, quality of length was the highest in central incisors (90.6%) followed by lateral incisors (83.3%) and canines (73.7%) while, the quality of density was the highest in first premolars (29.0%) followed by lateral incisors (16.7%), and the quality of taper was the highest in canines (42.1%) followed by lateral incisors (16.7%). However, in the mandible, central incisors had the highest quality of all criteria followed by second molars and then first molars (Table 5). Quality of root canal fillings according to root canal type is presented in Table 6. It can be noted that quality of length was most adequate in one canal roots (81.5%) followed by mesial roots (80.0%) and then distal roots (65.0%). Adequate density was found more in buccal roots (41.9%) followed by mesial roots (40.0%) and one canal roots (23.1%). However, adequate quality of taper was more frequent in one canal roots (34.3%) followed by buccal roots (25.8%) and mesial roots (20.0%).

**Table 5:** Quality of root canal fillings according to tooth type

	Length		Density		Taper	
	Inadequate	Adequate	Inadequate	Adequate	Inadequate	Adequate
Maxilla						
Central	3 (9.4)	29 (90.6)	28 (87.5)	4 (12.5)	27 (84.4)	5 (15.6)
Lateral	1 (16.7)	5 (83.3)	5 (83.3)	1 (16.7)	5 (83.3)	1 (16.7)
Canine	5 (26.3)	14 (73.7)	16 (84.2)	3 (15.8)	11 (57.9)	8 (42.1)
1st Premolar	19 (61.3)	12 (38.7)	22 (71.0)	9 (29.0)	29 (93.5)	2 (6.5)
1st Molar	64 (74.4)	22 (25.6)	85 (98.8)	1 (1.2)	83 (96.5)	3 (3.5)
2nd Molar	5 (83.3)	1 (16.7)	6 (100.0)	0 (0.0)	6 (100.0)	0 (0.0)
Mandible						
Central	11 (21.6)	40 (78.4)	34 (66.7)	17 (33.3)	28 (54.9)	23 (45.1)
1st Molar	58 (63.0)	34 (37.0)	89 (96.7)	3 (3.3)	89 (96.7)	3 (3.3)
2nd Molar	4 (50.0)	4 (50.0)	6 (75.0)	2 (25.0)	7 (87.5)	1 (12.5)
	**P < 0.001**		**P < 0.001**		**P < 0.001**	

Boldface refers to significance at *P* value < 0.05

**Table 6:** Quality of root canal fillings according to root canal type

	Length		Density		Taper	
	Inadequate	Adequate	Inadequate	Adequate	Inadequate	Adequate
Mesial (*n* = 5)	1 (20.0)	4 (80.0)	3 (60.0)	2 (40.0)	4 (80.0)	1 (20.0)
Distal (*n* = 100)	35 (35.0)	65 (65.0)	83 (83.0)	17 (17.0)	88 (88.0)	12 (12.0)
Palatal (*n* = 123)	60 (48.8)	63 (51.2)	96 (78.0)	27 (22.0)	101 (82.1)	22 (17.9)
Buccal (*n* = 31)	16 (51.6)	15 (48.4)	18 (58.1)	13 (41.9)	23 (74.2)	8 (25.8)
One canal (*n* = 108)	20 (18.5)	88 (81.5)	83 (76.9)	25 (23.1)	71 (65.7)	37 (34.3)
Mesiolingual (*n* = 95)	48 (50.5)	47 (49.5)	92 (96.8)	3 (3.2)	91 (95.8)	4 (4.2)
Mesiobuccal (*n* = 187)	108 (57.8)	79 (42.2)	180 (96.3)	7 (3.7)	174 (93.0)	13 (7.0)
Distobuccal (*n* = 92)	57 (62.0)	35 (38.0)	89 (96.7)	3 (3.3)	84 (91.3)	8 (8.7)
Total (*n* = 741)	345 (46.6)	396 (53.4)	644 (86.9)	97 (13.1)	636 (85.8)	105 (14.2)
	**P < 0.001**		**P < 0.001**		**P < 0.001**	

Boldface refers to significance at *P* value < 0.05

## Discussion

This study was carried out to assess the technical quality of root canal fillings performed by preclinical undergraduate dental students at the Faculty of Dentistry, Thamar University. To the best of our knowledge, this is the first study conducted among Yemeni dental students for this aspect. The material of this study consisted of the radiographs taken during the preclinical training of 4th year undergraduate dental students in the academic year 2016/2017. Radiographic interpretation of periapical disease is affected by the levels of inter-examiner variability and intra-examiner reproducibility [22–25]. In our study, the high inter-examiner reproducibility values supported a high level of reliability of the evaluation. The overall quality of the evaluate teeth fulfilled the three criteria was only 3.6% which is much lower than other previous studies [8, 11, 14, 18, 20, 26, 27]. These differences in the results might due to the variation in the evaluation criteria where some studies used only two criteria (length and density) [26, 28–31], most were conducted among clinical undergraduate students [8, 18, 20, 32–35], and others evaluated only one group of teeth [3, 12, 36, 37]. The curriculum of Endodontology in our dental school is given in two academic years (4 semesters). The first semester in the 4th years is a preclinical training course where the dental students trained how to perform RCT on human extracted teeth (single- and multi-rooted teeth) fixed in teaching models. Three hour per week are assigned for these requirements. This short time alongside with the low staff-to-students ratio plays important role for this result. Moreover, the stress and anxiety in the dental environment as well as the current conflict and violation in Yemen might result in low academic performance among dental students [38]. However, the overall quality of length in the present study was 53.4%, which is higher than that reported by Lupi-Pegurier eta al [39], who reported a length quality of 39% while, our result is lower than that reported by Balto et al. [35], Er et al. [8], Chuech et al. [40], and Eleftheriadis & Lambrianidis [7]. Although with no significant difference, the quality of length in the mandible was higher than that in the maxilla. This result is similar to that reported by Moradi & Gharechahi [28] while, it contradicts the results reported by Elsayed et al. [41], AbuMostafa et al. [34], and Elemam et al. [18], who reported higher quality in the maxilla with significant difference. These variations might due to the difference in the evaluation criteria, teaching methods, and/or type of the study sample (clinical vs. preclinical). Our study sample was radiographs of extracted teeth whereas the study samples in previous studies were radiographs of treated patients. The presence of tongue, saliva, and being the mandible a movable jaw may explain the higher quality in the maxilla among these studies.

As expected, the adequate length in the anterior teeth was higher than that in the posterior with highly significant difference. This result is similar to other studies [7, 35, 42]. However, it is not the case in the study conducted by Elsayed et al. [41], who reported higher quality in the posterior teeth than in the anterior. Similarly, adequate length of single-rooted teeth was higher than multi-rooted teeth with highly significant difference. Although there is scarce in the literature regarding studies dealt with the difference between single- and muli-rooted teeth, our result can compared to that of Roman-Richon et al. [14], Lynch & Burke [3], Er et al. [8], Khabbaz et al. [43], and Barrieshi-Nusair [44], who reported that teeth with less number of roots had higher quality than teeth with higher number of roots. Quality of length by type of tooth in the maxilla decreased gradually from central incisor toward molars teeth. This result is similar to that of Lynch and Burke [3], who reported gradual decrease of quality from centrals to premolars. Similar results were also reported by Er et al. [8] and Khabbaz et al. [43]. For mandibular teeth, we had central incisors showed a quality of 78% which is similar to that reported by Er et al. [8] and Lynch & Burke [3], who reported a quality of 74 and 78%, respectively. Quality of length of second molars was higher than that of first molars which contradicts the results of Lynch & Burke [3], who reported higher quality of first molars. However, this result is close to that reported by Balto et al. [35] and higher than that reported among Sudanese dental students [45]. Regarding quality of length according to canal location, our results revealed that inadequate length was more frequent in distobuccal and mesiobuccal canal. To our knowledge, there is no published paper in the literature compared the adequacy of root fillings in relation to canal location. Hence, comparison with other results is not valid. Proper knowledge of canal location and anatomy is very important factor in filling quality. Curvature of roots in these locations leads to some difficulties in preparation and instrumentation which result in decrease of filling adequacy.

The frequency of inadequate density of the treated teeth was much lower than adequate quality. This result is comparable to that reported by AbuMostafa et al. [34] but, contradicts many of previous studies. However, when this result related to tooth type, Er et al. [8], Saatchi et al. [31], and AlRahabi et al. [11] reported inadequate density of maxillary and mandibular molars higher than that of premolars and anterior teeth which is similar to our results with difference in proportions. Er et al. [8] also reported inadequate quality close to adequate quality of incisors and canines. In the current study, inadequate quality of RCT in maxilla was higher than in mandible with no significant difference. This result is similar to that reported by Elsayed et al. [41] and contradicts that of Saatchi et al. [31] who reported higher voids in mandible. Multi-rooted teeth showed higher problems in density than single-rooted teeth which is in agreement with the study conducted by Roman-Richo et al. [14] who found that density problems increased with the increase of in number of canals.

In relation to shaping quality, similar to density problems, the taper inadequacy of RCT was much lower than adequate taper. This result is contradicts many previous studies. However, inadequate taper was higher in maxilla than in mandible which is similar to that reported by Saatchi et al. [31] and Elsayed et al. [41]. Taper quality by tooth location and number of roots showed that posterior teeth and multi-rooted teeth had the highest frequency of tapering inadequacy. These results are close to those reported by Er et al. [8], AlRahabi et al. [11], Elsayed et al. [41], AbuMstafa et al. [34], and Roman-Richon et al. [14]. By tooth type, tapering problems were higher in molars teeth which similar to some previous studies [11, 18, 34]. By root canal location, mesiolingual, mesiobuccal, and distobuccal canals were the most subjected to shaping errors. Although no previous studies explored in details the RCT quality by root canal location, Eleftheriadis & Lambrianidis [7] reported that most iatrogenic errors (ledges) were found in these canals.

The RCT in undergraduate level was performed using the step-back technique by stain-less steel K-files. However, the sequential steps from apical-to-coronal can cause procedural accidents (ledges, canal transportation, perforation), resulting in ineffective root canal obturation. Of course, the introduction of Ni-Ti rotary instruments to endodontic practice revolutionized the cleaning and shaping of the root canal system. Due to their flexibility, Ni-Ti rotary systems cause less canal transportation and alteration of working length (WL) than do stainless steel instruments. So, the endodontics curriculum should thus be modified to cover advances in instruments and materials.

Potential limitations of the used method should be acknowledged. The retrospective study design limits the available information to that coming from the radiographic record base. Moreover, the inherent limitations of radiographic examination and interpretation may have introduced methodological errors. In particular, the radiographs were not taken in a strictly standardized and reproducible manner. Changes in beam and film angulation affect the radiographic appearance of the evaluated parameters. Bucco-lingual root canal curvatures, as well as procedural errors, may not always be accurately depicted on periapical radiographs. For example, a short filling may be a result of either a ledge or apically packed dentin chips and debris. Additionally, it has been shown that there is limited correlation between the radiographic appearance of the root canal filling and its adaptation and compaction [46]. Finally, the lack of phantom heads limited the extent of the potential to simulate clinical conditions. Few studies of the technical quality of RCT by preclinical students have been performed. It will be of great interest to conduct a research at the clinical level for the students of the same academic year to monitor the performance of their endodontic work and to compare their preclinical and clinical achievements.

## Conclusion

The technical quality of RCT performed by undergraduate dental students was found to be inadequate. This finding suggests that the training course in endodontics has to be improved at the preclinical level. It is recommended that new techniques and instruments incorporated into the curriculum to enhance the quality of endodontic practice.

## Data Availability

The datasets supporting the findings of this article are available from the corresponding author.

## Abbreviations

RCT: Root Canal Treatment
WL: Working Length
ZOE: Zinc Oxide Eugenol
